# Do Clonal Plants Show Greater Division of Labour Morphologically and Physiologically at Higher Patch Contrasts?

**DOI:** 10.1371/journal.pone.0025401

**Published:** 2011-09-30

**Authors:** Zhengwen Wang, Yuanheng Li, Heinjo J. During, Linghao Li

**Affiliations:** 1 State Key Laboratory of Forest and Soil Ecology, Institute of Applied Ecology, Chinese Academy of Sciences, Shenyang, China; 2 State Key Laboratory of Vegetation and Environmental Changes, Institute of Botany, The Chinese Academy of Sciences, Beijing, China; 3 College of Ecology and Environmental Science, Inner Mongolia Agricultural University, Huhhot, China; 4 Plant Ecology and Biodiversity, Institute of Environmental Biology, Utrecht University, Utrecht, The Netherlands; University Copenhagen, Denmark

## Abstract

**Background:**

When growing in reciprocal patches in terms of availability of different resources, connected ramets of clonal plants will specialize to acquire and exchange locally abundant resources more efficiently. This has been termed division of labour. We asked whether division of labour can occur physiologically as well as morphologically and will increase with patch contrasts.

**Methodology/Principal Findings:**

We subjected connected and disconnected ramet pairs of *Potentilla anserina* to Control, Low, Medium and High patch contrast by manipulating light and nutrient levels for ramets in each pair. Little net benefit of inter-ramet connection in terms of biomass was detected. Shoot-root ratio did not differ significantly between paired ramets regardless of connection under Control, Low and Medium. Under High, however, disconnected shaded ramets with ample nutrients showed significantly larger shoot-root ratios (2.8∼6.5 fold) than fully-lit but nutrient-deficient ramets, and than their counterparts under any other treatment; conversely, fully-lit but nutrient-deficient ramets, when connected to shaded ramets with ample nutrients, had significantly larger shoot-root ratios (2.0∼4.9 fold) than the latter and than their counterparts under any other treatment. Only under High patch contrast, fully-lit ramets, if connected to shaded ones, had 8.9% higher chlorophyll content than the latter, and 22.4% higher chlorophyll content than their isolated counterparts; the similar pattern held for photosynthetic capacity under all heterogeneous treatments.

**Conclusions/Significance:**

Division of labour in clonal plants can be realized by ramet specialization in morphology and in physiology. However, modest ramet specialization especially in morphology among patch contrasts may suggest that division of labour will occur when the connected ramets grow in reciprocal patches between which the contrast exceeds a threshold. Probably, this threshold patch contrast is the outcome of the clone-wide cost-benefit tradeoff and is significant for risk-avoidance, especially in the disturbance-prone environments.

## Introduction

Plants adopt multiple strategies to adapt to their natural environments, where one or more necessary resources always limit their growth. The best known one of these strategies is optimal allocation pattern, which means that plants should adjust allocation so that their growth is equally limited by all resources [1]–[3]. In a heterogeneous environment, however, adjacent and connected ramets within a plant clone are very likely growing in different microsites between which the availability of growth-limiting resources is negatively correlated [4], [5]. These ramets are predicted to allocate proportionally more biomass to structures responsible for acquiring resource(s) being abundant in their microsites but scarce for other connected ramets, and to exchange the acquired resources [6]–[9]. Such specialization and cooperation in clonal plants has originally been analogized to spatial division of labour in economic systems, and hereafter has been so termed [7].

Since plants are more effective in acquiring the abundant resources, division of labour between connected clonal ramets, which are negatively associated in availability of light and any edaphic factors, is believed to confer advantages on whole clonal fragments or entire clonal plants (genets), and hence to allow the plants to grow better [8]–[10]. This has been experimentally shown for *Trifolium repens* [7,8], *Fragaria chiloensis* [5], [6], [11], [12], *Potentilla anserina* [13], and *P. reptans* [14], *Glechoma hederacea* [15], [16], *G. longituba* [17], [18] and *Schoenoplectus americanus* [19]. These studies clearly showed the high potential benefits of division of labour to enhance resource capture of clonal plants and thereby to increase their performance in heterogeneous habitats compared to non-clonal plants.

One of the prerequisites for division of labour is adjacent patches with a certain degree of contrast between them in terms of availability of different types of resources [20]. Such habitats are ubiquitous under field conditions, as the availability of essential resources may be inherently negatively correlated, especially when a high availability of one resource tends to reduce that of another [6], [7], [21]. For example, nitrogen-fixing shrubs may increase soil nitrogen availability but decrease light levels under their canopies [5], [6], [22]. Such habitats are characterized by patch contrast, which is defined as the degree of relative difference in resource availability between patches or between a patch and its surrounding matrix [23]. A certain level of patch contrast is believed necessary to drive inter-ramet resource translocation [24]–[26], and may thus form an environmental constraint for division of labour.

Theoretical studies predicted that greater patch contrast in a patchy habitat could lead to stronger physiological integration [27], [28] or division of labour [29]–[31]. However, experimental evidence is rather scarce. Stuefer and Hutchings failed to detect division of labour between connected ramets of *Glechoma hederacea* exposed to either lightly or heavily contrasted patches in terms of light and nutrient availability [21]. They attributed the lack of any resource transfer to the absence of a water potential gradient between reciprocal patches, which would constrain transport of nutrients between ramets. In the same species, Wijesinghe and Hutchings showed that the connected ramets were more highly specialized and thereby gained greater total biomass when subject to more contrasted microsites in terms of nutrient availability, which can be interpreted only by the benefit of physiological integration [16]. Roiloa *et al.* showed that division of labour was stronger in clones originating from habitats of greater patch contrast than in those from more uniform habitats, but this was likely due to genotypic variation [5]. Thus, evidence for the positive relationship between environmentally-induced division of labour among clonal ramets and the patch contrast is far from conclusive.

Compared with morphological traits, physiological ones usually respond faster and more reversibly [10], [32], and thus are better indicators of functions of plant organs at a more refined time scale. Both chlorophyll content and photosynthetic capacity can show shade acclimation and light-foraging responses [33] and can be promoted by physiological integration of nitrogen [34] or enhanced by assimilate demand from connected ramets in shade [35]. Similarly, the ability of root systems to take up water or nutrients depends not only on their sizes [36], [37], but also on their uptake capacity per unit root mass [38], [39]. Root viability is one of the critical factors accounting for the uptake capacity of plant roots [40]. Therefore, chlorophyll content, photosynthetic activity and root viability promise to provide crucial information about the capacity for light and soil resource harvest, which may declare the physiological specialisation of plant parts. However, surprisingly few studies on division of labour have addressed physiological specialisation. In the only such study we know of, Roiloa *et al.* showed in greenhouse that ramets of *Fragaria chiloensis* were specialised in chlorophyll concentration in the same direction as in shoot proportional mass and leaf area [5]. This suggests that different traits may be functionally coordinated when responding to the same factors. Accordingly, if division of labour occurs, we expect that the connected ramets exposed to high light but low nutrient levels would have higher chlorophyll contents and photosynthetic capacities, but lower root viability.

To test the hypothesized positive relationship between the magnitude of division of labour and patch contrast, we conducted a garden experiment, in which connected and disconnected ramet pairs of a typical stoloniferous clonal plant, *Potentilla anserina* L., were subjected to a homogeneous treatment (Control) and three heterogeneous treatments with different patch contrasts (Low, Medium and High). One ramet in each pair was un-shaded and the other was well supplied with nutrients regardless of patch contrasts, but they were nutrient-stressed and shaded respectively, to different extent, depending on the patch contrast to which they were assigned. We predicted that in heterogeneous treatments: (i) The connected ramet pairs will achieve a greater total biomass than severed ramets, as a benefit of division of labour; (ii) Fully-lit but nutrient-stressed ramets will have larger shoot-root ratio (S/R), leaf areas, chlorophyll contents and photosynthetic capacity, but smaller root viability, when they are connected than when disconnected to the shaded but well-fed ramets, while the reverse pattern is true for the latter; (iii) Within the connected ramet pairs, fully-lit but nutrient-stressed ramets will show larger values of S/R, leaf area, chlorophyll content and photosynthetic capacity, but lower root viability, than their connected ramets in opposite patches, while the reverse pattern is true for the severed ramet pairs; (iv) Whether connected or not, the relative differences in above-mentioned parameters between within-pair ramets will increase with patch contrast.

## Methods

### Species and propagation

*Potentilla anserina* L. is a stoloniferous clonal herb with rosette-forming ramets that can produce sympodial, plagiotropic stolons with roots and leaves (i.e., potentially independent ramets) at nodes that touch moist soil during the growing season. Internodes are approx. 5∼15 cm long, depending on environmental conditions and on their positions along the stolons. The plant is common in grazed grasslands, road verges and sometimes grows with tall grasses [4], [41], where negative spatial covariance of resources often occurs. For instance, in grasslands, relatively low light intensity under tall grasses or shrubs usually associates with relatively high availability of water and nutrients due to the release from plant litter; while high light intensity without grasses and shrubs is usually accompanied by relatively low soil nutrient level and low water availability [4], [5].

The precultivation and the experiment were conducted in the yard of Duolun Restoration Ecological Research Station, Institute of Botany, Chinese Academy of Sciences (DRERS-IBCAS). In the yard, the sunlight intensity at daytime during the experimental period was up to 2000 µmol·m^−2^·s^−1^. On June 5 of 2007, we selected a healthy and vigorous rosette of *P. anserina* growing in the yard of DRERS-IBCAS to be the original genet for propagation. Dozens of plastic pots filled with sterilized river sand were placed around the rosette, to receive the daughter ramets. We supplied all the ramets with deionized water twice a day to keep soil moist, and additionally provided the parent ramet with a solution of a special fertilizer, Peters Professional (Pot Plant Special with N∶ P∶ K = 15∶10∶30. Plant growth is extremely well with this formula). On July 12, altogether more than 30 primary and secondary stolons were produced, and along each of them, four to nine offspring ramets had been produced. These offspring ramets were sufficient for selection of experimental ramet pairs.

### Experimental design

On July 12 of 2007, we selected 56 pairs of adjacent daughter ramets from the propagated clone, and cut them off the rest of the clone. From the rest, we selected 14 ramets ranging in size in terms of number and length of compound leaves, and then for each ramet we measured the length and the width for every compound leaf, and then determined area and weight for them. Based on these data, we got a regression model relating the dry weight of compound leaves to their length and width (Leaf weight = 0.0079 (leaf length×leaf width) ^0.7753^, n = 38, *R^2^* = 0.9085, *P*<0.0001). Prior to the experimental treatment application, we measured the length and width of all the leaves for each ramet within each standardized experimental ramet pair, and then according to the above-mentioned regression model, we calculated the shoot mass of the experimental ramets and thus ramet pairs as initial size. All the selected ramets were of similar size in terms of shoot biomass (One-way ANOVA, *F*_15, 96_ = 0.66; *P* = 0.8190). The two interconnected ramets of each pair were planted in paired adjacent pots (310 ml in volume, the pot size was justified by a pilot experiment conducted in 2006) filled with sterilized river sand. Half of these ramet pairs were disconnected by cutting the stolon connection halfway between the paired ramets, thus resource transfer was impeded and therefore division of labour was prevented. The other half were left intact. Both connected and disconnected ramet pairs were further randomly assigned to four patch contrasts increasing from Control, as the only homogeneous treatment, to Low, Medium and High as three heterogeneous treatments, giving rise to seven replicates for each treatment.

The scheme for light exposure and nutrient application for all the ramets involved in the experiment is shown in Table 1. In the homogeneous treatment, both ramets were un-shaded and supplied with 50 ml 0.1500 mg·ml^−1^ solution of Peters Professional once every five days, giving rise to null patch contrast with highest light and nutrient availability. In the three heterogeneous treatments, we shaded one ramet within each pair by covering it with polypropylene shade cloth of different shading intensities, while reducing nutrient availability for the other simply by decreasing the concentration of fertilizer solution (Table 1). On the days when none of the ramets were to be fertilized, we applied 50 ml deionized water to all experimental ramets twice with the intention to keep soil moist. As a result, a patch contrast gradient was formed, which intensified from Control to Low, Medium and High patch contrast in terms of two types of resources: light and soil nutrients, being captured by shoots and roots, respectively. The pilot experiment suggested that nutrient status can be kept relatively stable (i.e., nutrient accumulation can be avoided in some sense) in the pots filled with sterilized river sand if they were fertilized and watered in a regime as used for our experimental pots.

**Table 1:** Light exposures and nutrient concentrations applied to the ramets in homogeneous (Homo: C) and heterogeneous (Hetero: L, M and H) treatments during the experiment.

		HL-VN		VL-HN	
	Patch contrasts	Light (% full sunlight)	Nitrogen (mg/ml)	Light (% full sunlight)	Nitrogen (mg/ml)
Homogeneous	Control	100	0.1500	100	0.1500
Heterogeneous	Low	100	0.0750	50	0.1500
Heterogeneous	Medium	100	0.0375	20	0.1500
Heterogeneous	High	100	Water only	5	0.1500

Notes: The fertilizer used is Peter Professional, in which N∶ P∶ K = 15∶10∶30. The solution of fertilizer (and water only) was applied as 50 ml once every 5 days. See text for further information.

For the sake of description, we coded the unshaded ramet in each ramet pair HL-VN (always exposed to high light but varying nutrient levels) and coded the other VL-HN (always exposed to high nutrient level but varying light exposure) in the heterogeneous treatments. Such codes were randomly assigned to the two ramets of each pair in the homogeneous treatment (Control). To detect if direction of the ramet pairs mattered, for four of the seven replicates of each treatment, the proximal (developmentally older) ramets were assigned as VL-HN and the distal (developmentally younger) ones as HL-VN, and hereafter this direction of ramet pair was referred to as YO; while the other three replicates were positioned otherwise, and hereafter the direction was referred to as OY.

### Measurements

We measured chlorophyll contents using a portable chlorophyll meter (SPAD-502, Konica Minolta Sensing, Inc., Tokyo, Japan) three times with an interval of 15 days from 13 August onwards. Only four replicate ramet pairs for each treatment, where the proximal ramets were assigned as VL-HN and the distal ones were assigned as HL-VN, were selected to measure photosynthetic rates using the Photosynthesis Analyzing System (Li-6400, Li-Cor). The measured leaves, always the same ones as those measured for chlorophyll content, were illuminated at 1500 µmol m^−2^ s^−1^ using the LED light system. We harvested the experiment nine weeks after the treatment application. When harvesting, we separated the shoots and roots, and directly measured total leaf area for each ramet by scanning with Epson Perfection V200 Photo Scanner, and subsequent image analysis. Afterwards, for ramet pairs whose photosynthetic rates had been determined, we determined the root viability using the improved triphenyl tetrazolium chloride (TTC) reduction technique [42]. All the shoots and roots were then dried at 70° for 48 h to measure their biomass and S/R was calculated.

### Data Analyses

The effects of patch contrast, connection, direction of ramet pairs and their interactions on total biomass, S/R and leaf area of HL-VN and VL-HN ramets were analyzed with three-way ANOVAs. The same analysis was also performed for the total biomass of whole ramet pairs. Because only the ramet pairs in which younger ramets were assigned as HL-VN had been measured for root viability, two-way ANOVAs were used to test the effects of patch contrast, connection and their interaction on root viability of the ramets. Duncan's multiple range tests were performed to test the differences in total biomass of ramets, S/R, leaf area and root viability between HL-VN ramets and between VL-HN ramets. To detect the advantage of stolon connection for entire ramet pairs, we used Student's *T*-test to compare the total biomass of ramet pairs between connected and disconnected treatments. Repeated-measure ANOVAs were used to test the effects of patch contrast, connection, direction of ramet pairs and their interactions on chlorophyll content and photosynthetic capacity (direction not applicable) over time separately for HL-VN and VL-HN. Separately for each patch contrast, repeated-measure ANOVAs were also used to detect the effect of direction on the chlorophyll content of HL-VN, which was found to be affected by direction in the three-way ANOVA. The effects of patch contrast on the relative difference between HL-VN and VL-HN in chlorophyll content and photosynthetic capacity were also analyzed using repeated measure ANOVAs separately for severed and connected treatments. The relative differences between paired ramets were used as dependents to allow for the comparison among different patch contrasts and were calculated as the differences between the two ramets divided by their sum.

Paired *T*-test was performed to test the differences in S/R, chlorophyll content index, photosynthetic capacity, leaf area and root viability between paired ramets at all patch contrasts. Using repeated measure ANOVAs, the differences in chlorophyll content and in photosynthetic capacity over time between severed and connected treatments were tested separately for HL-VN and VL-HN ramets at each patch contrast. All the statistical analyses were performed with SAS 9.1.2 [43]. Data of ramet biomass, S/R and leaf area were log-transformed to meet homoscedasticity before ANOVA was performed.

## Results

### Biomass and shoot-root ratio

**Figure 1:**
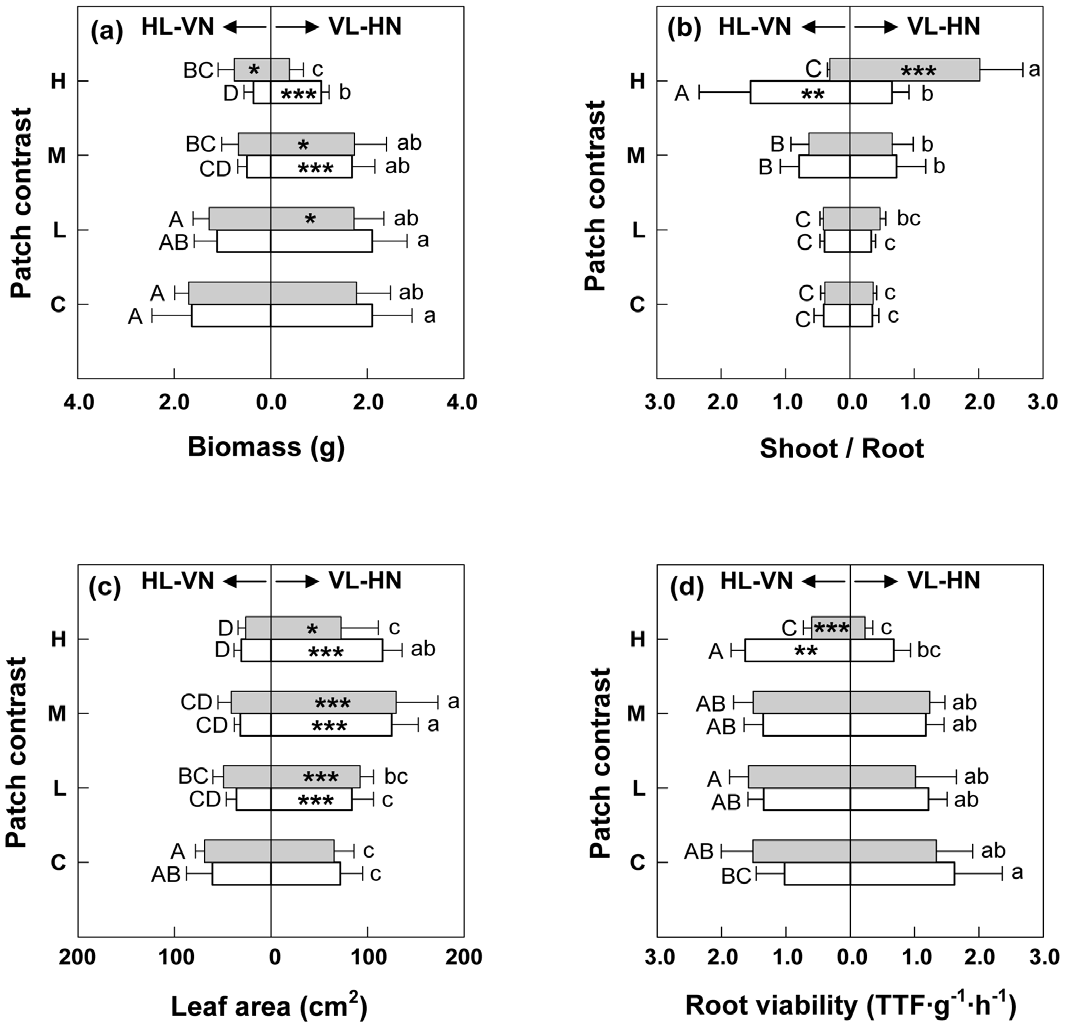
Total biomass (a), shoot-root ratios (b), leaf area (c) and root viability (d) of ramets. Open bars and closed bars are for connected and severed treatments respectively. The capital letters in the left and the lower case letters in the right are significance test results for difference between treatments for HL-VN ramets and VL-HN ramets respectively. C, L, M and H on the vertical axes stand for Control, Low, Medium and High patch contrast, respectively. The treatments with the same letter are not significantly different at P = 0.05. The asterisks on the bars denoted the paired student *T*-test results for difference between the paired ramets. *: 0.01<*P*<0.05; **: 0.001<*P*<0.01; ***: *P*<0.001.

**Figure 2:**
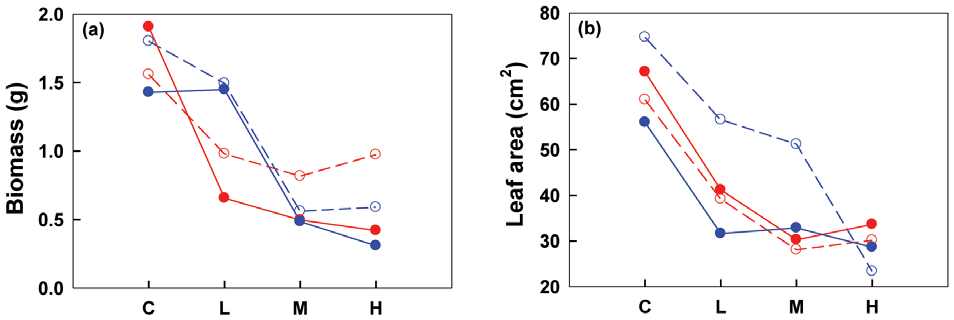
Total ramet biomass (a) and leaf area (b) of HL-VN. C, L, M and H on the horizontal axes stand for Control, Low, Medium and High patch contrast, respectively. In the panels, the red dashed lines with open circle symbols and red solid lines with closed circle symbols stand for OY direction of severed and connected treatments, respectively, while the blue dashed lines with open circle symbols and blue solid lines with closed circle symbols stand for YO direction of severed and connected treatments, respectively. See the text for the definition of OY and YO direction.

For the main effects, total biomass of individual ramets decreased with the increase of patch contrast, and was affected by connection only at High, while that of the whole ramet pairs was only affected by patch contrast (Table 2; Fig. 1a; Fig. 2a). Direction itself did not show any effects on the total biomass of individual ramets or the entire ramet pairs. The interactive effect of PC×Connection on the total biomass of VL-HN suggested that the response of VL-HN biomass to patch contrast was affected by connection. Similarly, the interactive effect of PC×Direction on the total biomass of HL-VN and the whole ramet pair suggested that their responses to patch contrast were affected by direction of ramet pairs (Table 2; Fig. 1a; Fig. 2a). However, repeated measure ANOVAs showed that the total biomass of HL-VN differed significantly in response to direction only at Low (YO>OY). In the homogeneous treatment, disconnection showed no effects on ramet growth. Whether connected or not, VL-HN showed little difference in biomass among Control, Low and Medium, but became significantly lower under High. Isolated HL-VN decreased gradually in biomass with increasing patch contrast, and the connected ones showed smaller sizes than the isolated ones under High. Unexpectedly, total biomass of the connected ramet pairs did not differ significantly from that of disconnected pairs at any patch contrast (Table 2; Fig. 1a). S/R of individual ramets was significantly affected by patch contrast, connection and their interaction, but not by direction, nor by any other interactive effect (Table 2; Fig. 1b). Paired *T*-tests revealed that S/R did not differ significantly between paired ramets regardless of connection under Control, Low and Medium. Under High, however, VL-HN, when isolated from HL-VN, had significantly larger S/R (2.8∼6.5 fold) than the latter and than its counterparts under all the other treatments; conversely, HL-VN, when connected to VL-HN, had a significantly larger S/R (2.0∼4.9 fold) than the latter and than its counterparts under all the other treatments (Fig. 1b).

**Table 2:** Three-way ANOVAs for the effects of patch contrast, connection, direction of ramet pairs and their interactions on total biomass, S/R, leaf area and root viability of ramets and total biomass of ramet pairs.

Factors	d.f.	HL-VN		VL-HN		Ramet pair	
		*F*	P	*F*	P	*F*	P
Total biomass							
Patch contrast (PC)	3	20.11	**<0.0001**	20.82	**<0.0001**	27.69	**<0.0001**
Connection	1	8.35	**0.0062**	12.18	**0.0015**	0.89	0.3504
Direction	1	0.12	0.7309	0.18	0.6733	0.28	0.6009
PC×Connection	3	1.44	0.2452	5.11	**0.0018**	0.72	0.5439
PC×Direction	3	3.64	**0.0207**	2.30	0.0918	3.05	**0.0394**
Connection×Direction	1	0.13	0.7208	0.55	0.4628	0.52	0.4760
PC×Connection×Direction	3	0.56	0.6452	1.44	0.2443	0.71	0.5524
Error	40						
S/R							
Patch contrast (PC)	3	11.74	**<0.0001**	27.02	**<0.0001**		
Connection	1	23.31	**<0.0001**	13.98	**0.0006**		
Direction	1	1.45	0.2364	0.67	0.4174		
PC×Connection	3	18.55	**<0.0001**	7.67	**0.0004**		
PC×Direction	3	0.60	0.6164	1.09	0.3664		
Connection×Direction	1	0.49	0.4903	0.02	0.8943		
PC×Connection×Direction	3	0.24	0.8710	0.56	0.6473		
Error	40						
Leaf area							
Patch contrast (PC)	3	15.88	**<0.0001**	6.06	**0.0017**		
Connection	1	2.61	0.1142	2.48	0.1234		
Direction	1	0.20	0.6609	0.45	0.5070		
PC×Connection	3	1.49	0.2329	2.09	0.1174		
PC×Direction	3	1.85	0.1544	1.29	0.2904		
Connection×Direction	1	5.58	**0.0232**	0.64	0.4277		
PC×Connection×Direction	3	0.88	0.4607	1.13	0.3502		
Error	40						
Root viability							
Patch contrast (PC)	3	2.00	0.1405	7.67	**0.0009**		
Connection	1	0.11	0.7431	1.93	0.1778		
PC×Connection	3	8.97	**0.0004**	0.45	0.7179		
Error	24						

Values of P<0.05 are in bold.

### Leaf area and root viability

Three-way ANOVAs showed that leaf area of individual ramets was affected only by patch contrast, not by connection or direction, but there was a Connection×Direction effect on the leaf area of HL-VN (Table 2). However, repeated measure ANOVAs declared no significant difference among different Connection×Direction combinations at any patch contrast except for that of severed YO at Medium, being larger than that of any other combination (Table 2; Fig. 2b). Unexpectedly, VL-HN developed a significantly larger leaf area than the corresponding HL-VN in all the heterogeneous treatments regardless of connection and disconnection of ramet pairs significantly affected leaf area of VL-HN only under High (Fig. 1c).

Root viability of VL-NH decreased significantly with patch contrast, and that of HL-VN was affected by patch contrast×Connection. Under Control, Low and Medium, there was little difference in root viability between ramets in all the cases regardless of connection, and connection showed no effects on root viability of either ramet in a pair. However under High, HL-VN had higher root viability than the corresponding VL-HN whether connected or not, and disconnection significantly decreased root viability of both VL-HN and HL-VN (Table 2; Fig. 1d).

### Chlorophyll contents

**Figure 3:**
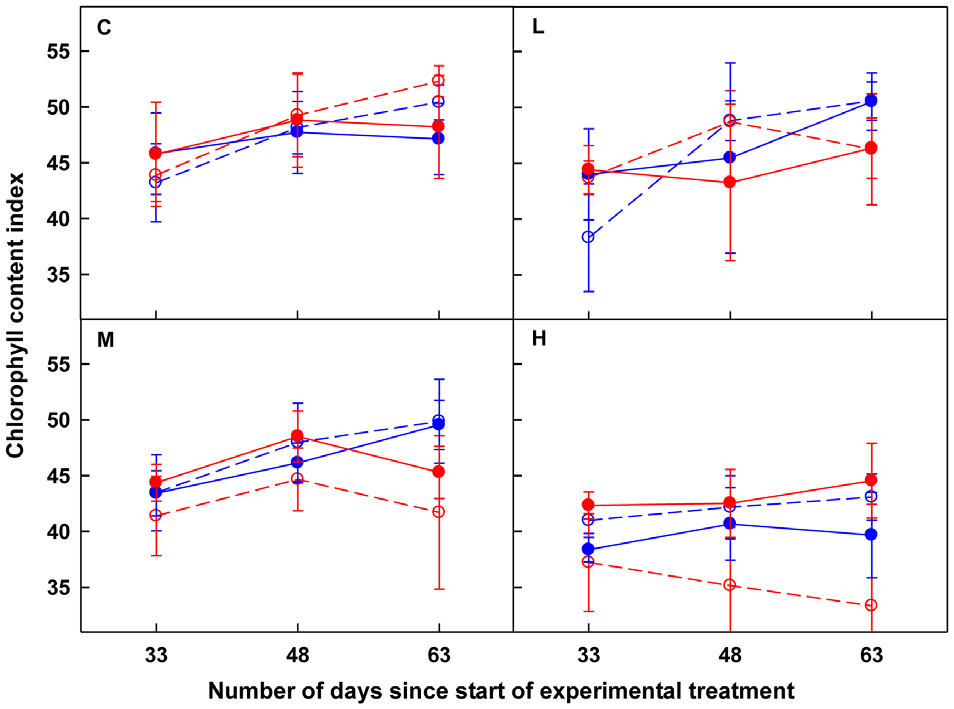
Chlorophyll content index of ramets. In the panels, the red dashed lines with open circle symbols and red solid lines with closed circle symbols stand for HL-VN ramets of severed and connected treatments, respectively, while the blue dashed lines with open circle symbols and blue solid lines with closed circle symbols stand for VL-HN ramets of severed and connected treatments, respectively. C, L, M and H inside the panels stand for Control, Low, Medium and High patch contrast, respectively. See table 3 and the text for significance of difference between treatments.

**Figure 4:**
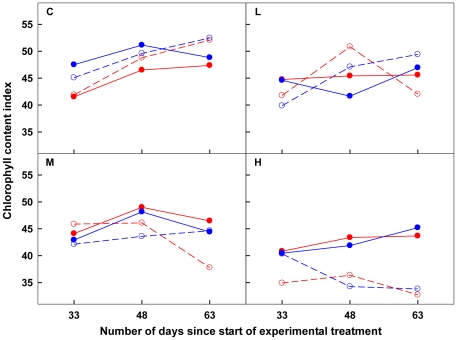
Effects of direction on Chlorophyll content index of HL-VN. In the panels, the red dashed lines with open circle symbols and red solid lines with closed circle symbols stand for OY direction of severed and connected treatments, respectively, while the blue dashed lines with open circle symbols and blue solid lines with closed circle symbols stand for YO direction of severed and connected treatments, respectively. C, L, M and H inside the panels stand for Control, Low, Medium and High patch contrast, respectively. See the text for the definition of OY and YO direction.

Repeated measure ANOVAs showed that chlorophyll contents of both HL-VN and VL-HN were significantly affected by patch contrast. Connection and patch contrast×connection had significant effects on chlorophyll contents of only HL-VN, but direction alone showed no effects (Table 3A; Fig. 3). Chlorophyll content displayed a significant time effect, and this effect could be affected by patch contrast. Connection and direction showed significant effects on the temporal dynamics of chlorophyll content of VL-HN and HL-VN, respectively. However, repeated measure ANOVAs separately done for each patch contrast showed that no main or interactive effect of direction was found on chlorophyll content of HL-VN except for a main effect at Control (*F_1,8_* = 5.85; *P* = 0.0419), being higher for YO than for OY (Fig. 4). The interaction of patch contrast and connection had significant effect on the temporal dynamics of chlorophyll content of individual ramets (Table 3A; Fig. 3). VL-HN had higher chlorophyll contents when detached from than connected to HL-VN at High (*F*_1, 12_ = 5.24; *P* = 0.0410); conversely, HL-VN had significantly higher chlorophyll contents when connected to than when isolated from VL-HN at Medium (*F*_1, 12_ = 5.67, *P* = 0.0347) and High (*F*_1, 12_ = 11.06, *P* = 0.0060) (Fig. 3). In the severed treatment, VL-HN had significantly higher chlorophyll contents than HL-VN over time at Medium (*F*_1, 12_ = 5.85, *P* = 0.0324) and High (*F*_1, 12_ = 8.67, *P* = 0.0123), while this pattern was reversed if they were connected at High (*F*_1, 12_ = 8.89, *P* = 0.0115) (Fig. 3). Repeated Measure ANOVAs also revealed that the relative differences within connected and isolated ramet pairs changed significantly with patch contrast (Table 4A; Fig. 3). On average of the three measurements, under High patch contrast, chlorophyll content of HL-VN being connected to VL-HN, was 8.9% higher than that of the latter, and 22.4% higher than that of isolated HL-VN.

**Table 3:** Repeated measures ANOVA for the effects of patch contrast, connection and their interaction on chlorophyll content index (A) and photosynthetic capacity (B).

Effects	d.f.	HL-VN		VL-HN	
		*F*	*P*	*F*	*P*
**(A) Chlorophyll content index**					
Between subjects effects					
Patch contrast (PC)	3	19.48	**<0.0001**	17.78	**<0.0001**
Connection	1	8.72	**0.0054**	0.88	0.3533
Direction	1	0.32	0.5750	0.21	0.6515
PC×Connection	3	5.60	**0.0029**	0.93	0.4350
PC×Direction	3	0.05	0.9865	1.78	0.1674
Connection×Direction	1	1.36	0.2518	1.41	0.2428
PC×Connection×Direction	3	0.20	0.8944	1.20	0.3239
Error	37				
Within subjects effects					
Time	2	8.05	**0.0007**	71.31	**<0.0001**
Time×PC	6	4.40	**0.0007**	6.10	**<0.0001**
Time×Connection	2	1.45	0.2402	7.66	**0.0009**
Time×Direction	2	7.17	**0.0014**	1.01	0.3686
Time×PC×Connection	6	3.11	**0.0091**	3.21	**0.0075**
Time×PC×Direction	6	0.95	0.4638	0.74	0.6197
Time×Connection×Direction	2	2.24	0.1133	0.13	0.8798
Time×PC×Connection×Direction	6	0.78	0.5895	0.37	0.8968
Error (time)	74				
**(B) Photosynthetic capacity**					
Between subjects effects					
Patch contrast (PC)	3	2.37	0.0968	73.00	**<0.0001**
Connection	1	14.33	**0.0010**	31.80	**<0.0001**
PC×Connection	3	1. 06	0.3868	1.06	0.3864
Error	23				
Within subjects effects					
Time	2	7.55	**0.0015**	30.82	**<0.0001**
Time×PC	6	3.20	**0.0104**	13.92	**<0.0001**
Time×Connection	2	1.45	0.2445	4.19	**0.0212**
Time×PC×Connection	6	1.33	0.2616	3.76	**0.0039**
Error (time)	46				

Values of *P*<0.05 are in bold.

**Table 4:** Repeated measures ANOVAs for the effects of patch contrast on the relative differences (RD) within ramet pairs in chlorophyll content index (A) and photosynthetic capacity (B).

Effects	d.f.	Severed		Connected	
		*F*	*P*	*F*	*P*
**(A) RD in Chlorophyll content**					
Between subjects effects					
Patch contrast	3	5.38	**0.0059**	3.79	**0.0248**
Error	23				
Within subjects effects					
Time	2	16.18	**<0.0001**	1.57	0.2198
Time×Patch contrast	6	2.51	**0.0345**	1.99	0.0881
Error (time)	46				
**(B) RD in Photosynthetic capacity**					
Between subjects effects					
Patch contrast	3	10.19	**0.0013**	45.52	**<0.0001**
Error	12				
Within subjects effects					
Time	2	16.41	**<0.0001**	7. 17	**0.0040**
Time×Patch contrast	6	1.80	0.1407	1.88	0.1296
Error (time)	24				

Values of *P*<0.05 are in bold.

### Photosynthetic capacity

**Figure 5:**
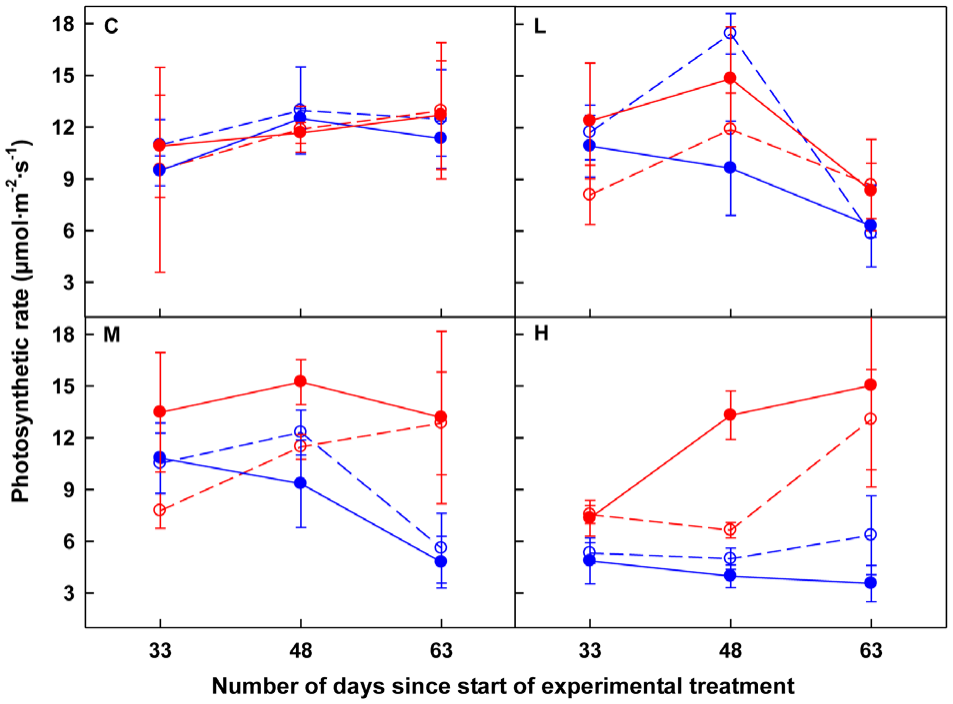
Photosynthetic capacity of ramets. In the panels, the red dashed lines with open circle symbols and red solid lines with closed circle symbols stand for HL-VN ramets of severed and connected treatments, respectively, while the blue dashed lines with open circle symbols and blue solid lines with closed circle symbols stand for VL-HN ramets of severed and connected treatments, respectively. C, L, M and H inside the panels stand for Control, Low, Medium and High patch contrast, respectively. See table 3 and the text for significance of difference between treatments.

Photosynthetic capacities of both HL-VN and VL-HN were affected by patch contrast (only marginally significant for HL-VN) and connection, and showed obvious temporal dynamics, which was also subject to patch contrast (Table 3B; Fig. 5). VL-HN showed greater photosynthetic capacity when disconnected than when connected to HL-VN in all the heterogeneous treatments (*F*_1, 6_ = 16.89, *P* = 0.0093; *F*_1, 6_ = 9.70, *P* = 0.0208; *F*_1, 6_ = 7.93, *P* = 0.0305 under Low, Medium and High, respectively), while the latter showed greater photosynthetic capacity when connected than when disconnected to the former at Medium (*F*_1, 6_ = 11.50, *P* = 0.0147) and High (*F*_1, 6_ = 9.25, *P* = 0.0228) (Table 3B; Fig. 5). At an early stage, isolated VL-HN tended to have a greater photosynthetic capacity than corresponding HL-VN ones once they were exposed to Low and Medium (Fig. 5). Instead, for the latest census at Medium and all those at High, HL-VN showed a greater photosynthetic capacity than corresponding VL-HN if they were isolated from each other (Fig. 5). However, in the connected treatment, HL-VN had greater photosynthetic capacity than VL-HN in all the three heterogeneous treatments (*F*_1, 6_ = 6.32, *P* = 0.0658; *F*_1, 6_ = 40.89, *P* = 0.0007; *F*_1, 6_ = 91.07, *P* = 0.0001 under Low, Medium and High respectively) (Fig. 5). Moreover, the relative differences in photosynthetic capacity between within-pair ramets changed drastically with patch contrast both for severed and connected pairs (Table 4B; Fig. 5).

## Discussion

### Patch contrast threshold for division of labour

The ramet biomass pattern at homogeneous treatment showed no severing effects on the growth of *Potentilla*. Benefit from stolon connection in terms of biomass accumulation was shown for VL-HN only under High, but not for HL-VN or for the entire pairs of ramets under any patch contrast. Nevertheless, S/R suggested that the ramet specialization characteristic of division of labour occurred under High. In a greenhouse experiment with *Fragaria chiloensis*, however, ramet specialization was observed under both low and high patch contrasts involved, and total biomass of connected ramet pairs also increased at high patch contrast [6]. Both cases encourage future efforts to ascertain whether and when division of labour increases total growth over a longer period especially in realistic heterogeneous habitats [9]. The biomass of isolated ramets across patch contrasts suggests that growth of VL-HN was limited by light only under High contrast (i.e., low light availability), and that of HL-VN by nutrients under Medium and High contrast. Thus, it is likely that morphological division of labour for light and nutrients will occur only when both types of resources are negatively associated in terms of their availability to the interconnected ramets and are limiting their growth.

The absence of ramet specialization or division of labour under relatively low patch contrasts may be related to the mechanisms of resource transfer. One recognized mechanism is based on the source-sink relationship, which assumes that differences in resource availability to interconnected ramets form internal gradients in resource abundance [25], [44]. The resource transfer will not take place until the resource gradient is strong enough to surmount the inherent resistance that baffles the resource movement. Moreover, resource transport brings costs due to energy consumption [9]. This has been corroborated by a recent model study showing that the two interconnected ramets exhibit division of labour if the benefit is larger than the costs of water transportation [31]. Accordingly, the resource gradients under Low and Medium in our study may not be strong enough for the plants to ultimately activate resource transfer.

Despite circumstantial evidence for reciprocal transport of assimilates and water between interconnected fragments of *Potentilla anserina* [7], [13], [14], nutrient transport is usually coupled to and thus constrained by the flow of water or assimilate [26]. However, since most nutrients are transported in the xylem [45], [46], nutrient sharing is likely to be effective in the presence of parallel gradients in water potential between nutrient sources and sinks [21]. In our experiment, the presence of such a gradient between interconnected ramets was quite likely in heterogeneous setup, because the evapotranspiration for VL-HN ramets was lower than for the corresponding HL-VN ramets due to their different light exposure [26]. Indeed, by our observation, the soil in the pots for HL-VN ramets was always drier than that for the corresponding VL-HN ramets except for immediately after water application. Once such water transport is taking place, however, the nutrient concentration in the xylem of the ramets in the high-nutrient patch may be expected to reflect the nutrient availability in the soil and thus affect the rate of nutrient transport via the stolons. Fortunately, in the present experiment, water could be seen as parallel to nutrient in view of the following facts: 1) water gradient was always directionally consistent with nutrient gradient; 2) both types of resources (water and nutrient) are soil factors which should be captured and taken up by same structures, say, roots; 3) theoretically, the intensity of the water gradient is positively associated with that of the nutrient gradient.

### Ramet specialisation in physiological features

The general pattern in chlorophyll contents of both the severed and connected ramet pairs confirmed the last prediction in the Introduction with respect to chlorophyll contents (Table 4A; Fig. 3). When disconnected, HL-VN showed reduced chlorophyll contents with increasing patch contrast due to increasing shortage of nitrogen, while VL-HN had to acclimate to shaded environments in the heterogeneous treatments, and thus were stimulated to produce more chlorophyll. It has been found that shaded plants would invest a greater proportion of nitrogen [47] into the light-harvesting machinery, which accounts for about half of the total chlorophyll [48]. This is a local plastic response expressed in chlorophyll. Basically the same pattern was found for the connected ramet pairs at Low, where paired ramets affected each other little since division of labour did not occur, as suggested by the biomass allocation pattern. At High, however, chlorophyll content of connected ramets clearly showed effects of division of labour, being higher in HL-VN than in VL-HN.

The patterns of photosynthetic capacity almost fully complied with our second prediction. Generally, the ramets had lower photosynthetic capacity at higher patch contrast, probably due to the decreased availability of nitrogen for HL-VN and of light for VL-HN (Table 3B; Fig. 5). Photosynthesis is strongly dependent on leaf nitrogen content and the incident light. Since in most plant species more than half of leaf nitrogen is allocated to photosynthetic proteins, a strong linear positive relationship exists between photosynthetic capacity and leaf nitrogen content [49], which is largely dependent on the external nitrogen available to the plants. On the other hand, leaves decrease in photosynthetic capacity when shaded [50], [51], because of reduced nitrogen allocated into the enzymes responsible for CO_2_-fixation, as a consequence of increased investment into the light-harvesting complex when acclimating to low irradiance [51], [52]. Despite that, the status of connection in our case did exert effects on photosynthetic capacity of ramets (Table 3B). As predicted, fully-lit connected ramets in the three heterogeneous treatments showed significantly greater photosynthetic capacities than isolated ramets in the same conditions. This pattern suggested that the connected HL-VN in the three heterogeneous treatments had been specialized to some extent for photosynthesis, e.g., by investing proportionally more nitrogen to the production of enzymes responsible for CO_2_-fixation [53].

Our findings confirm the idea that division of labour is actualized not only by adjusting biomass partitioning, but also by regulating certain physiological functions [5]. For the first time, we experimentally demonstrated ramet specialization in photosynthetic capacity characteristic of division of labour. Since physiological traits are more easily reversible than morphological traits [9], physiological specialization may be less risky for the entire clones or clonal fragments, especially in a temporally unstable context. For instance, physiological plasticity allows rapid increase in nutrient uptake capacity of roots in response to unpredictable short nutrient flushes especially in habitats with inherently infertile soils, and plant leaves can immediately promote their photosynthetic capacity when exposed to sunflecks interrupting periods of low light [2]. Thus, division of labour may be hypothesized to be more readily expressed in physiological traits (especially photosynthetic performance) at a more refined time scale than in morphological traits.

### Unexpected responses of leaf area and root viability

Unexpectedly, ramets exhibited typical local plastic responses regardless of connection in leaf area at all the heterogeneous treatments and in root viability under High contrast. Although the ramets showed division of labour in biomass allocation under the highest patch contrast, the responses of ramets in leaf area and root viability complied with optimal foraging theory. That is to say, the responses of leaf area and root viability were not in accordance with that of biomass allocation, chlorophyll content and photosynthetic capacity. Perhaps the patterns of leaf area and root viability follow the predictions of division of labour theory at even more pronounced patch contrast. This incongruity gives rise to a concept of division of labour as a syndrome of coordinated responses of multiple traits with each trait being characterized by its own response time and level of threshold patch contrast.

### Effects of direction

No main effects of Direction were found for any plant trait (Table 2, Table 3). Although three-way ANOVAs showed a significant effect of PC×Direction and Connection×Direction on the total biomass and leaf area of HL-VN respectively, more detailed analysis did not result in any coherent patterns across the four patch contrasts with respect to Direction. Repeated ANOVAs showed significant Time×Direction effects on the chlorophyll content of HL-VN, but the same analysis done for each patch contrast separately did not show any significant differences between the two directions for all the three heterogeneous treatments. Taken together, it was speculated that the directional constraint for the translocation of resources within clonal system of *Potentilla anserina* is negligible. Vascular constraint is unlikely for *Potentilla anserina* because bidirectional transport of assimilate and water had already been demonstrated [14]. Interconnected clone parts of *Potentilla anserina* can virtually share assimilates according to internal source-sink relationships, which also occurs in some other dicotyledonous species such as *Trifolium repens*, *Fragaria chiloensis* and *P. reptans* [26]. On the other hand, directional constraints are less likely to affect water transport in plants because it is a passive process driven by water potential gradients, which arise from intra-clonal differences in water loss and water uptake [26]. Thus, nutrient translocation, being dependent on intra-clonal gradients in assimilate and/or water supply, was little affected by direction of ramet pairs.

### Limitations

There are two limitations in the present experiment. Firstly, all the experimental ramet pairs originated from a single genotype of *Potentilla anserina*, as was done in a previous study on division of labour [21]. This limited the extrapolation of conclusions drawn from the present study, as genotypic variation in the degree of division of labour does exist, as recently shown in *Fragaria chiloensis* [5]. After all, our focus is to find out the effect of patch contrast on the magnitude of division of labour, as long as it can happen to a genotype. The other limitation is that the gradient of patch contrast is confounded with that of the overall resource level. The overall resource level available to ramet pairs in terms both of light and of nutrients decreased with increasing patch contrast. This prevents us from completely teasing apart the effects of the overall resource levels and their contrasts the ramet pairs experienced. However, assuming that the two ramets within each pair should basically behave the same if they were exposed to similar conditions regardless of overall resource level, the different shoot-root ratios between them under High contrast would be explained only by patch contrast.

### Conclusions

Division of labour in clonal plants can be realized by ramet specialization in morphology, but maybe more readily in physiology. Contrary to the intuitive assumption, division of labour (and its characteristic ramet specialization) will not occur unless the connected ramet pairs experience reciprocal and highly contrasted patches, where the growth of individual ramets is restricted. Accordingly, we hypothesized that division of labour occurs when the connected ramets grow in negatively associated patches between which the contrast in terms of resource abundance exceeds a threshold, and in which the focal resource is limiting the growth of local ramets. This threshold was indeed found, but was not consistent among different traits in terms of the magnitude of patch contrast under which the ramet specialization characteristic of division of labour in the traits occurred. Such trait-specific thresholds and modest levels of ramet specialisation in some traits may have been selected for as mechanisms to avoid risks of specialization especially in disturbance-prone environments. What should follow next will be efforts to test and generalize the above-mentioned hypothesis across genotypes and even among species, and simultaneously to tease apart the effects of overall resource levels available to and the contrast between the paired ramets on ramet specialisation or division of labour.
